# Comorbid Attention-Deficit/Hyperactivity Disorder and Mood Disorder in a South African Sample of Substance Use Disorder Patients

**DOI:** 10.3390/jcm14030927

**Published:** 2025-01-31

**Authors:** Ilse Truter, Judith Regnart, Anneke Meyer

**Affiliations:** Drug Utilization Research Unit (DURU), Department of Pharmacy, Faculty of Health Sciences, Nelson Mandela University, Gqeberha 6031, South Africa; judith.oettle@gmail.com (J.R.);

**Keywords:** attention-deficit/hyperactivity disorder (ADHD), co-occurring disorder (COD), mood disorder, substance use disorder (SUD), comorbidity, drug utilisation

## Abstract

**Background:** The brain reward circuitry is thought to underlie the co-occurrence of attention-deficit/hyperactivity disorder (ADHD) and substance use disorder (SUD) and to possibly impact mood disorders. This study aimed to establish if any difference existed in the severity of depression symptomology between SUD comorbidity with and without ADHD. **Methods**: A multi-centre, cross-sectional comparison study design drew study participants from substance use treatment facilities within South Africa. The participants were screened for ADHD and depression, with the selective application of a confirmatory ADHD diagnostic interview. The participants were diagnostically grouped according (SUD + ADHD, SUD − ADHD) to an application of a 2 x 2 x 3 ANOVA model. **Results**: A significant main effect of ADHD diagnosis and gender on depressive symptoms was identified. Post hoc analysis revealed that only male ADHD subjects had significantly higher scores on the Beck scale than non-ADHD males. **Conclusions**: Co-occurring disorder (COD) prevalence rates were higher than most other South African studies. The aggravation of ADHD on mood disorder symptom severity is consistent with the existing literature; however, further investigation is warranted to determine if the interaction of gender remains only significant for men with a lager sample size. The identified COD prevalence rate may contrast with other South Africa studies, emphasising the need for comprehensive psychiatric comorbidity screening in SUD treatment settings.

## 1. Introduction

Epidemiological investigations in recent decades have increasingly acknowledged the overlap of substance use disorder (SUD) with psychiatric conditions [1], noting that patients with such presentations are at greater risk of delayed diagnosis, more severe symptoms, reduced treatment response and impaired adherence, greater social functioning impairment, suicidal ideation, homelessness, and even criminal activity [2,3]. It has been demonstrated that SUD, when presenting with comorbid psychiatric conditions, is associated with a more severe presentation of substance use, characterised by the adolescent onset of SUD with rapid progression between first substance use and the emergence of disordered usage [4].

The existing literature also provides evidence to support the overlap of the co-occurrence of SUD with ADHD and mood disorders within substance-abusing populations. One or more SUDs are reported to be identified in up to 20% of individuals with mood disorders [5] and approximately 50% of individuals with ADHD [6]. In the investigation of patients attending a private rehabilitation centre in South Africa by Fabricius and colleagues [3], co-occurring (psychiatric) disorders (CODs) were identified in 57.1% of the study population, with over 65% of these patients being diagnosed with a mood disorder and 16% with ADHD. Another South African study reported a substance use prevalence of 50% in a sample of adolescents attending psychiatric services [7]. In the study by Chan and colleagues [4], an assessment of the pooled prevalence of internalising and externalising disorders reported in substance abuse treatment studies revealed that in the past year, the prevalence of depression varied from 32.7% in young adolescents to 56.2% in adults over the age of 40 years, while ADHD prevalence exceeded 30% in all reported age groups. The meta-analysis by van Emmerik-van Oortmerssen and colleagues [8] determined the mean prevalence of ADHD in SUD populations to be 23.1%. Similar results were reported for a study conducted in Cairo [9], with screening for ADHD identifying potential diagnosis in between 15.69% and 17.65% of the sample depending on the screening instrument applied. A cross-sectional study, which included multiple sites across ten countries [10], reported the prevalence of ADHD in treatment-seeking SUD populations to range from 7.6% to 32.6%, while a study from another international cohort [11] reported that most participants had previously received treatment for SUD, but a great portion of those participants were treatment-naïve for ADHD. This study also reported that 61.4% of the sample presented with at least one other psychiatric comorbidity over and above SUD [11]. 

The South African Community Epidemiology Network on Drug Use (SACENDU) was established by the Medical Research Council of South Africa (MRC) in 1996 through funding from the World Health Organization (WHO) and is a sentinel surveillance system which is operational in all nine provinces of South Africa [12,13]. SACENDU’s bi-annual reports on data from July 2014 through to December 2018 refer to the term dual diagnosis, with such rates reported upon admission for SUD treatment ranging from 13% to 19% [14,15,16,17,18,19,20,21,22]. Of the 19% of dual diagnosis subjects referenced in SACENDU’s research brief published at the time of this study, 41% were reported to be mental illness comorbidities [22]. Given that these statistics were based on reports upon admission and not indicative of subsequent diagnoses while receiving treatment, the true rate of mental illness comorbidity within South African substance-using populations is estimated to be higher based on the existing international and South African literature [14,15,16,17,18,19,20,21,22].

With consideration for the known neurobiological underpinnings, together with the stated epidemiological data, the aim of this study was to establish the difference in severity of depression symptomatology between SUD comorbidity with ADHD and SUD without ADHD treatment-seeking patients. The following objectives were identified that we sought to achieve:

- To screen for the presence of ADHD and mood disorder in SUD patients;
- To quantify depression symptom severity;
- To confirm a diagnosis of ADHD;
- To determine the point prevalence of different CODs within a substance-using population.

## 2. Materials and Methods

A multi-centre, cross-sectional comparison study design made use of convenience sampling, with the study participants being drawn from six substance use treatment facilities within South Africa’s Gauteng and Eastern Cape provinces. A target of 200 participants was envisioned for the study population. Van de Glind and colleagues [10] also sampled patients from different sites in their study, with similar sample sizes. The participants were adults over the age of 18 years attending in-patient treatment facilities for SUD, with the applied exclusion criteria being a history of head injury or non-completion of primary school-level education.

### 2.1. Data Collection Instrumentation

Data collection consisted of two stages, each including the collection of specific information from participants with the application of validated psychometric tools:

1. Screening phase:
• Demographic information (e.g., age, gender, ethnicity, first language);
• Adult ADHD Self-Report Scale (ASRS) version 1.1;
• Beck Depression Inventory (BDI).

2. Diagnostic phase:
• Diagnostic Interview for ADHD in adults (DIVA).

#### 2.1.1. The Adult ADHD Self-Report Scale (ASRS) Vers 1.1

The ASRS is designed for the collection of ADHD symptoms in the context of adulthood by relating symptoms to adult situations such as work, tasks, or projects [23]. This reliable and valid self-administered instrument was jointly developed in 2005 by the WHO and Kessler and colleagues [24]. The ASRS vers 1.1 consists of 18 questions which are based on the criteria used for diagnosing ADHD in the DSM-IV-TR (Text Revision) [24]. Six of these items—the ASRS Part A (ASRS-A)—have been found to be most predictive of symptoms consistent with a diagnosis of ADHD [24].

#### 2.1.2. Beck Depression Inventory

The Beck Depression Inventory (BDI) is one of the most extensively used measures for the measurement of the intensity of depression in psychiatric populations and detection of depression in normal populations [25,26]. The instrument was derived from clinical observations of the attitudes and symptoms often displayed by depressed psychiatric patients, with systematic consolidation into 21 symptoms and attitudes [25]. These items were included to assess the intensity of depression as they are based on its main symptoms, meaning that the tool is generally considered to have high content validity [26]. It is noted that although the tool was designed to be administered by trained interviewers, it is most often self-administered [25]. Despite the BDI being developed as a screening tool, it can also be applied to confirm a diagnosis of depression [27].

#### 2.1.3. Diagnostic Interview for ADHD in Adults (DIVA 2.0)

The DIVA 2.0 interview allows for a thorough evaluation of the DSM-IV-TR criteria for ADHD in adulthood [28]. It is divided into two domains to assess symptoms applicable to childhood (before age 12 years) and adulthood, while the third part considers functional impairment in five areas of functioning in both age categories [28]. The study conducted by Ramos-Quiroga and colleagues [28] reported that the DIVA 2.0 has similar psychometric properties to what is considered to be the gold standard diagnostic interview for adult ADHD (Conners’ Adult ADHD Diagnostic Interview for DSM-IV [CAADID]), while good correlation was found with the Wender Utah Rating Scale (WURS) for evaluating childhood symptoms. Overall, the authors concluded that it was a reliable diagnostic tool with good predictive value for the diagnosis obtained [28].

### 2.2. Data Collection Process

**Figure 1:**
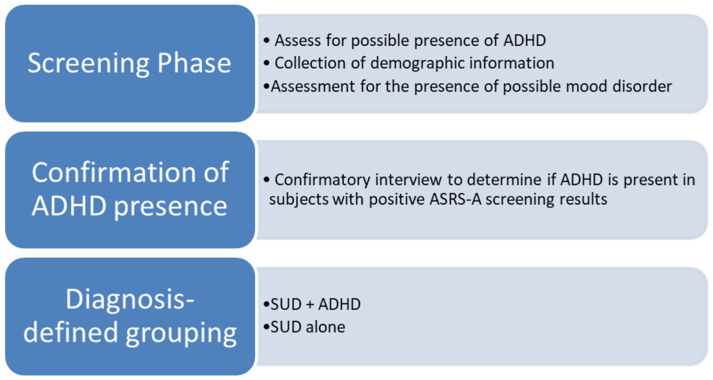
Summary of the data collection process.

The primary stage of the data collection consisted of screening, in which the possible presence of adult ADHD and mood disorder was assessed. The secondary, diagnostic phase involved all participants who screened positively for ADHD based on the ASRS-A results being interviewed, with the application of the Diagnostic Interview for ADHD in Adults (DIVA) to confirm the presence of ADHD. Following this, the participants were grouped according to diagnoses: SUD with ADHD (SUD + ADHD) and SUD without ADHD (SUD − ADHD). This process is illustrated in Figure 1. Data were collected between July 2018 and June 2019 by three study colleagues within the different regions.

### 2.3. Ethical Approval

Permission to conduct this study at each of the treatment facilities was arranged individually via the respective management entities with consideration for the previously granted ethical approval for the investigation from the Nelson Mandela University Research Ethics Committee (Human) (H14-HEA-PHA-081) on 11 December 2014. The study participants were provided with a document describing the investigation processes. This was accompanied by the present researcher(s) giving a general address to describe the processes and applied instruments with the opportunity for questions from the participants to be answered. Informed consent was obtained from the participants prior to completion of the data collection tools.

### 2.4. Statistical Analysis

Data were analysed using Statistica v. 12 (Dell Software). A 2 x 2 x 3 (ADHD group x gender x age group) ANOVA model was employed to indicate between-group differences. Post hoc (Bonferroni) analysis was employed to reveal within-group differences.

## 3. Results

Screening for ADHD produced a total of 153 subjects; however, ADHD diagnosis could not be verified in 5 of the subjects. A total of sample size of 148 participants was thus achieved, with diagnostic stratification represented as follows: SUD + ADHD, n = 45 and SUD − ADHD, n = 103.

The study participants represented eight of South Africa’s nine provinces. The greatest provincial representation was for Gauteng (43.24%, n = 64), followed by the Eastern Cape (34.46%, n = 51), Limpopo (6.76%, n =10), Mpumalanga (6.08%, n = 9), KwaZulu-Natal (2.70%, n = 4), Western Cape (2.70%, n = 4), North West Province (2.03%, n = 3), and Free State (2.03%, n = 3). The participant home language endorsement revealed that all of South Africa’s 11 official languages were included in the study sample, the greatest proportion of participants being Afrikaans-speaking (31.76%, n = 47), followed by Xhosa (18.24%, n = 27), English (15.54%, n = 23), and Zulu (10.81%, n = 16).

More than half of the study participants endorsed African ethnicity (53.38%, n = 79), and the study population was predominantly male (79.05%, n = 117). A mean age of 31.76 ± 10.08 years was obtained, with the female portion of the study population being slightly older, on average at 33.68 ± 8.63 years against 31.25 ± 10.37 years for men. Further demographic considerations are depicted in Table 1. Table 2 indicates the result of the ANOVA.

There was a significant main effect of ADHD diagnosis: F(1, 135) = 12.45, *p* < 0.001, ηp2 = 0.08 and a significant main effect of gender: F(1, 135) = 4.78, *p* = 0.03, ηp^2^ = 0.03 on depressive symptoms, as indicated by the scoring of the Beck scale.

There was no effect of age, neither main nor interacting.

The post hoc analysis (Bonferroni) revealed that only the male ADHD group (M = 25.81, SD = 11.59) had significantly higher scores than the non-ADHD males (M = 16.34, SD = 10.95) on the Beck scale: *p* < 0.001.

The difference in the scores between the female ADHD group (M = 31.36, SD = 11.91) and the non-ADHD group (M = 21.12, SD = 11.42) did not differ significantly: *p* = 0.08, although it approached significance.

## 4. Discussion

Our target study sample size was not met; however, the achieved sample of 148 subjects was considered adequate. Ethnic representation, viewed together with the inclusion of all the official languages of South Africa, was considered favourable, although it diverged from the 2019 mid-year population estimates for South Africa, with over- and disproportionate representation of Caucasian and mixed-race participants [29]. This disproportional representation could be due to historical inequality within South Africa as a result of its former apartheid regime, with it being noted by Myers and colleagues [30] that historically disadvantaged groups may experience limited access to substance abuse treatment. With respect to ADHD prevalence within the sample as a function of ethnicity, it was noted that only 16.85% (n = 89) of the African participants were confirmed to have ADHD against 51.11% (n = 45) of the Caucasian participants, a finding which could be construed to infer a lower prevalence of ADHD within this ethnic division. However, it has previously been reported that although the ASRS has been shown to have adequate internal consistency within a multi-cultural South African population sample, problems were experienced with relating the English tool to indigenous-African-language speakers [31]; as such, it is considered that culturally related condition inferences based on the ethnic representation reported in this investigation should be interpreted with caution. 

It was noted that a greater number of participants endorsed the completion of matric (grade 12) and post-matric (tertiary) education within the non-ADHD group; however, the relative proportions were higher for the ADHD group in these categories. This result was statistically significant (*p* = 0.01). This was not anticipated given that the presence of ADHD is typically associated with lower education levels [32]. However, given that the study sample was drawn from substance-abusing populations, associated with cognitive impairment [33], it is perhaps not remarkable. 

This sample was notably predominated by men, representing 79.05% of the total study population (N = 148). SUD has been described as a “male-dominated” disorder, with men making up over 70% of substance users, a finding replicated in the study by Arfken and colleagues [34]. With consideration for the existing literature indicating greater tendencies towards impulsivity in men, as evidenced by a higher prevalence rate of impulse-control-related conditions such as ADHD [32], greater expression of impulsivity as an atypical symptom of depression, and a higher rate of suicide [35] against the established theory of SUD being associated with heightened impulsivity derived from abnormal functioning of the brain reward circuitry [36], it is perhaps expected that SUD should be more prevalent in men. However, it is also posed that research in substance use populations has been historically skewed towards the outcomes in men [37]. Despite lower prevalence rates of SUDs in women, women presenting for SUD treatment are reported to experience more severe problems [37]. It is noted that 83.87% (n = 31) of the women in this study sample were identified as having CODs against 68.38% (n = 117) of the male participants, possibly reflecting such trends of women experiencing greater psychiatric problems upon referral or admission for SUD treatment [37]. It is proposed that the reasons for this phenomenon are multifactorial, including more financial barriers to accessing treatment, reduced availability of time to attend treatment, and a greater susceptibility to stigmatisation, culminating in treatment avoidance with associated exacerbation of existing conditions [37].

The presence of ADHD was found to be associated with greater mean depressive symptom severity, with post hoc analysis revealing that this was only significantly so for male participants. It was noted that this finding approached significance for the female participants, it being contemplated that a significant association might have been identified had the sample size been larger. Further to the interaction of ADHD on severity of mood disorder experienced by the study participants, it was also noted that the prevalence of ADHD in the study population was skewed towards co-presentation with mood disorder; 86.67% (n = 45) of the participants with ADHD also experienced mood disorder. Previous investigation into the comorbid presentation of ADHD and mood disorder has been without resolve for the determination of the extent to which this presentation is due to either genetic or environmental factors [6]. However, with both conditions being highly impairing, it has been shown that the course of depression in the presence of ADHD is associated with an earlier age of onset of depression, longer durations of depressive episodes, higher rates of suicidality, greater risk, and poorer long-term outcomes [6,38]. It is considered that this interplay, particularly with the increase in suicidality, which further implicates increased impulsivity, may also infer the involvement of the brain reward circuitry given its role in eliciting euphoria and anhedonia, with such extremes of mood being implicated in mood disorder symptomology [39]. It is noted that the study by Howard and colleagues [40] determined that depression and ADHD appear to be independent, additive sources contributing to substance use risk.

With consideration for the prevalence rates for CODs reported in other South African data, it was noted that the identified COD rate in the study population was over three times greater than the highest rate reported by SACENDU [22] and 14.5% higher than the rate reported by Fabricius and colleagues [3]. These findings support the speculation that the identification of CODs is under-recognised in South African SUD populations, emphasising the need for appropriate screening and surveillance incorporating the associated results to allow for improved prevalence reporting.

The influence of ADHD on mood disorder severity identified in this study is consistent with the existing literature; however, further investigation is warranted to determine if the interaction of gender within the ADHD group remains only significant for men. A key consideration relates to the contrasts identified with respect to COD prevalence rates in South Africa, emphasising the need to undertake comprehensive screening for psychiatric comorbidities in SUD treatment settings. Further investigation incorporating such activity may provide an improved view of the extent of CODs and their potential influence on SUD treatment outcomes.

The findings of this study should be considered against the following limitations:

1. Diagnostic screening was limited to ADHD and mood disorder; thus, there is potential for other comorbid psychiatric disorders to have been present in the study population.
2. No confirmatory procedure was applied to test the validity of the positive mood disorder screening. The decision to omit such activity was related to mood disorders being routinely screened for psychiatric settings and the potential application of the BDI as a diagnostic tool.
3. Diagnoses elicited as a result of the applied instrumentation were not confirmed by a treating physician at the facilities sampled.
4. The application of validated psychometric tools was considered appropriate for the purposes of the study methodology; however, the application of such tools in a population where English was not the most commonly reported first language may limit the reliability of the results.
5. Over-representation of men in the study population is noted to have an impact on the generalisability of the findings.

## 5. Conclusions

The prevalence rates of CODs in this study may indicate a gap in SUD assessment for CODs. There is, therefore, a need to incorporate screening practises to identify CODs in these populations, especially given the potential exacerbation of CODs on overall outcomes for SUD, as well as further between-condition interactions in the presence of more than one COD, as was identified in this investigation. It should, however, be noted that means of incorporating such screening tools may be challenging to apply in practice and that, as stated in the study limitations, their reliability within the South African context may be limited due to the wide variation in languages spoken and cultural influences. More targeted research to broaden the applicability of validated psychometric tools for such populations is recommended.

## Figures and Tables

**Table 1:** Demographics of the sample. * *p* < 0.05; ** *p* < 0.001.

	ADHD n = 45 (30%)		No ADHD n = 103 (70%)		ꭕ^2^
	n	%	n	%	*p*
**Age**					0.53
18–29	20	13.5	48	32.4	
30–40	15	10.1	40	27.1	
41+	10	6.8	15	10.1	
**Gender**					0.02 *
Male	31	20.9	86	58.1	
Female	14	9.5	17	11.5	
**Ethnicity**					<0.001 **
African	55	15.3	134	37.2	
Coloured	11	3.1	22	6.1	
White	61	16.9	72	20.0	
Indian	3	0.8	2	0.6	
**Home language**					0.09
Afrikaans	21	14.2	26	17.6	
English	10	6.8	13	8.8	
Sepedi	2	1.4	6	4.1	
Xitsonga	1	0.7	2	1.4	
Tshivenda	0	0.0	2	1.4	
isiXhosa	5	3.4	22	14.9	
isiZulu	2	1.4	14	9.6	
Setswana	3	2.0	6	4.1	
Sesotho	1	0.7	7	4.7	
siSwati	0	0.0	2	1.4	
isiNdebele	0	0.0	2	1.4	
**Employment**					0.27
Employed	23	15.8	38	26.0	
Unemployed	14	9.6	46	31.5	
Part-time	3	2.1	4	2.7	
Self employed	0	0.0	2	1.4	
Student	5	3.4	7	4.8	
Pensioner	0	0.0	4	2.7	
**Education level**					0.01 *
Primary	0	0.0	2	1.4	
Secondary	9	6.2	85	24.2	
Matric	20	13.8	30	20.7	
Post-matric	16	11.0	21	14.5	

**Table 2:** Analysis of variance (ANOVA). * *p* < 0.05; ** *p* < 0.001.

	DF	** F **	*p*	Partial Eta-Squared
**ADHD Diagnosis**	1, 135	12.45	<0.001 **	0.08
**Gender**	1, 135	4.78	0.03 *	0.03
**Age Group**	2, 135	0.63	0.53	0.01
**ADHD x Gender**	1, 135	0.03	0.87	0.00
**ADHD x Age Group**	2, 135	1.96	0.14	0.03
**Gender x Age Group**	2, 135	0.74	0.48	0.01
**DIVA x Gender x Age Group**	2, 132	0.43	0.65	0.01

## Data Availability

The dataset presented in this article is not available due to the ethical agreement signed by the university. Requests to access the dataset should be directed to the chairperson of the university’s Research Ethics Committee.

## Abbreviations

The following abbreviations are used in this manuscript:

ADHD	Attention-deficit/hyperactivity disorder
CD	Conduct disorder
COD	Co-occurring disorder
MDD	Major depressive disorder
ODD	Oppositional defiant disorder (ODD)
SUD	Substance use disorder
